# Estimation of Fine-Grained Foot Strike Patterns with Wearable Smartwatch Devices

**DOI:** 10.3390/ijerph19031279

**Published:** 2022-01-24

**Authors:** Hyeyeoun Joo, Hyejoo Kim, Jeh-Kwang Ryu, Semin Ryu, Kyoung-Min Lee, Seung-Chan Kim

**Affiliations:** 1 Interdisciplinary Program in Cognitive Science, Seoul National University, Seoul 08826, Korea; hyes@snu.ac.kr (H.J.); kminlee@snu.ac.kr (K.-M.L.); 2 Machine Learning Systems Laboratory, Department of Sports Science, Sungkyunkwan University, Suwon 16419, Korea; hyejoo98@g.skku.edu; 3 Department of Physical Education, College of Education, Dongguk University, Seoul 04620, Korea; ryujk@dongguk.edu; 4 Intelligent Robotics Laboratory, School of Artificial Intelligence Convergence, Hallym University, Chuncheon 24252, Korea; sr@hallym.ac.kr

**Keywords:** healthcare wearables, deep sequence learning, fine-grained motion classification, activity monitoring, human activity recognition

## Abstract

People who exercise may benefit or be injured depending on their foot striking (FS) style. In this study, we propose an intelligent system that can recognize subtle differences in FS patterns while walking and running using measurements from a wearable smartwatch device. Although such patterns could be directly measured utilizing pressure distribution of feet while striking on the ground, we instead focused on analyzing hand movements by assuming that striking patterns consequently affect temporal movements of the whole body. The advantage of the proposed approach is that FS patterns can be estimated in a portable and less invasive manner. To this end, first, we developed a wearable system for measuring inertial movements of hands and then conducted an experiment where participants were asked to walk and run while wearing a smartwatch. Second, we trained and tested the captured multivariate time series signals in supervised learning settings. The experimental results obtained demonstrated high and robust classification performances (weighted-average F1 score > 90%) when recent deep neural network models, such as 1D-CNN and GRUs, were employed. We conclude this study with a discussion of potential future work and applications that increase benefits while walking and running properly using the proposed approach.

## 1. Introduction

As interest in healthcare has increased; research on human activity recognition (HAR) is also receiving increasing attention. Smart devices offer affordable and less invasive methods for monitoring such daily activities. For example, most modern smartwatches offer advanced health-tracking features. With recent advances in machine learning techniques, human activities are recognized in a more precise and robust way [1,2,3,4,5]. Walking and running have a high proportion in every facet of daily activities; thus, these two activities have often been studied as key elements in the process of HAR in the past decades [2,6,7,8,9,10,11,12]. Owing to the repetitive nature of these activities, it is generally known that proper walking and running styles are crucial in simultaneously securing player safety and performance.

In the case of running, the rear-foot (RF) strike pattern associated with greater vertical loading [13] and larger impact peak forces [14] have been related to the cause of athlete injuries, such as tibial stress syndrome, plantar fasciitis, tibial stress fractures, patellofemoral pain, and Achilles tendon injuries [14,15,16]. Furthermore, a previous study proved that the FS patterns are closely related to the destruction of red blood cells, so-called hemolysis, compared to foot strike-free sports due to the mechanical trauma of foot strike on the ground [17]. Thus, RF runners with forceful landings on the ground are at a higher risk of hemolysis [18]. In this vein, although arguable [19], changing FS patterns from RF strike pattern to non-RF strike pattern, that is, midfoot (MF) or forefoot (FF), is generally recommended to avoid such injuries associated with running [13,20].

In the case of walking, the FS patterns also significantly affect the whole-body dynamic posture. For example, young children who are about to learn to walk usually toddle when walking, owing to their toe-to-heel (i.e., FF strike) walking strategy [21]. Similarly, people with pathological conditions, such as cerebral palsy, muscular dystrophy, stroke, autism spectrum disorder, and chronic pain, normally show FF strike pattern [22,23], so that they, consequently, have a waddling gait pattern.

Therefore, recognizing the striking patterns of running and walking would provide a meaningful translation of the quality of these physical activities to users, including athletes. Considering that the physical impact during contact with the foot is propagated through the whole body while walking and running, we hypothesized that the type of walking and running patterns (i.e., FS patterns) can be recognized from body parts other than the foot (e.g., wrist). Measuring the type of FS pattern in a natural and noninvasive manner is challenging because it normally requires dedicated equipment, such as a force plate or floor-mounted force platform [24,25]. Recent smart insoles offer portable alternatives for recognizing FS pattern; however, it may be inconvenient for some users, especially in a situation where activity needs to be monitored naturally in the presence of an insole. This may make users feel different or even uncomfortable while exercising. In addition, smart insole sensors can be contaminated with sweat or dust and are likely to be damaged by physical pressure from the ground and feet. In this context, we propose an intelligent wearable system that can recognize subtle differences in walking and running styles in the form of FS pattern in a portable and less invasive manner by leveraging the sensing capability of modern smartwatches.

To validate our approach, we first conducted experiments in which the participants were asked to walk and run with different striking patterns. Multivariate time-series data (MTS) were collected from a smartwatch using a custom software. We then evaluated whether captured motion signals reveal enough information to differentiate FS patterns in a supervised learning setting by utilizing recent deep learning architectures [26], including gated recurrent neural networks (RNNs) (e.g., long short-term memory (LSTM) [27], gated recurrent units (GRUs) [28]), and one-dimensional convolutional neural networks (Conv1D) [29,30]. In this study, conventional feature-based machine learning algorithms along with extensive feature engineering processes were employed to measure the baseline performance levels. The experimental results demonstrated that the proposed approach could recognize different types of walking and running styles with inertial measurements from the hand. In summary, this study primarily contributes to the literature in the following two ways:

1. With the hypothesis that different types of ground contact during walking and running would result in differences in whole-body movements, we propose an intelligent system that can indirectly observe and recognize the FS patterns based on MTS signals measured from a smartwatch.
2. We conducted two experiments (i.e., walking and running) to validate the proposed approach, which aims to investigate whether captured motion signals from the wrist deliver enough information to differentiate FS patterns.

In the next section, we will briefly review the relevant work.

## 2. Related Work

Human activity recognition (HAR) plays a significant role in various fields, including healthcare, surveillance, and human-computer interaction applications. Among the modalities of sensor-based approaches [31], we mainly focus on body-worn sensors throughout this study because these wearable sensors are ubiquitous (e.g., smartphones [6,8,9,32,33], smartwatch/band [32,34,35], and generic embedded devices [12,36,37,38]), and, thus, are unobtrusively applicable to users who exercise.

### 2.1. Human Activity Recognition

An earlier pioneering work proposed a wearable system that recognized a variety of daily activities using multiple embedded sensors [12]. By exploiting a variety of acceleration feature values, the system successfully recognized a set of everyday human activities, such as walking, sitting, bicycling, working on a computer, riding an escalator, etc. The experimental results also suggested that the use of multiple sensors aided the recognition of complex activities. With advances in mobile and wearable computing technologies, the number of HAR studies that utilize smart devices has increased accordingly. For example, in a previous study, Kwapisz et al. proposed an activity recognition system based on cell phone accelerometers that utilized the sensing capability of smartphones [33]. Their system could recognize six different physical activities using the 10-s of activity segmentations. In a recent study, Reyes-Ortiz et al. further extended the activity recognition capabilities by predicting not only a specific activity but also the transition between activities [39].

Recent advances in deep learning have enabled fine-grained motion analysis, which is challenging because similar activities normally require the extraction of subtle differences in motion features. For example, a recent study proposed the use of a wearable smartwatch device-based system for recognizing various hand-oriented everyday activities [34]. Their experimental results validated the feasibility of using the smartwatch measurements to differentiate similar activities, such as shaving and putting on lipstick with one’s hands. In a recent work, Haque et al. proposed an attention-based recurrent neural network that recognized six different nursing activities [40]. They achieved reasonable classification results by utilizing the proposed location and motion features. Another study presented an attention-based neural network system for recognizing user activities. By utilizing the CNN and GRU along with the attention model, the proposed system could learn both spatial and temporal dependencies from the complex multivariate time-series motion signals [41]. More recently, we formulated a classification problem for a variety of walking patterns, such as regular walking, walking while carrying objects (e.g., dumbbell, mobile phone, and umbrella) and walking with different gestures (e.g., walking with arms crossed, walking with hands behind the back, walking with hands in pockets, etc.) as a supervised machine learning problem [42]. Remarkably, it was found that human hand motion delivered sufficient information to recognize subtle differences between walking contexts.

### 2.2. Recognition of Foot Motions from Other Body Parts Apart from Feet

All body parts are organically connected; therefore, the movement of one part of the body can be transmitted to another. This human characteristic has often been exploited as a method to ensure natural interaction or additional modality, especially in the field of human-computer interaction. For example, in a previous study, Scott et al. proposed an interaction system that recognized fine-grained foot gestures, such as ankle dorsiflexion, plantar flexion, and heel and toe rotations, utilizing measurements from a sensor located in a pocket. Because foot motion subsequently moves the lower body parts, though subtly, a sensor located even in a pocket on the upper body parts (e.g., near the waist) could capture this motion [8].

Similarly, wearable sensors have recently been used to recognize gait patterns to detect certain medical symptoms. For example, a previous study proposed a wearable system consisting of multiple accelerometers mounted around the waist to analyze gait patterns. Their experimental results validated the feasibility of using gait features obtained from body-worn sensors for diagnosing cognitive declines [43].

### 2.3. Sensor-Based Recognition of Foot Strike Patterns

Efforts have been made to recognize different fine-grained running styles (i.e., foot strike patterns) through the use of wearable devices. For example, a recent study proposed a wearable system that explored pressure-sensitive insoles incorporated in the normal running shoes to predict foot-strike patterns while running. Experimental results demonstrated a high recognition rate (over 90%), although precise recognition of the MF strike pattern was found to be more challenging than those of FF and RF strikes [44]. Similarly, another recent work proposed a foot-striking recognition system based on a pressure-measuring sensor mounted inside a running shoe [45]. The experimental results revealed that the FS patterns were accurately recognized, similar to those reported previously [44]. Contrary to previous studies [44,45], that used pressure-measuring wearable insoles, another recent study utilized an accelerometer inside a running shoe to recognize two different landing styles (e.g., RF and FF strikes) while running [46]. The study found that a cross-correlation measure between acceleration signals from different axes could be used as a feature to recognize the foot strike patterns and the study envisioned the use of acceleration signals for foot strike classification while running.

Although measuring pressures [44,45] and inertial motions [46] of the foot or leg can directly provide information regarding foot strike patterns, we hypothesized that such information can also be observed in body parts other than the feet or legs, such as the hands, since the human body parts are connected organically; thus, different loading patterns while striking on the ground would result in significant differences in whole-body motions. From this perspective, we utilized a wearable smartwatch as a sensing device to observe and recognize the hand motion affected by foot strike patterns. To prove hypothesis, we developed a smartwatch-based wearable system that can recognize detailed running and walking patterns, such as FF, MF, and RF striking patterns. We then evaluated whether the captured motion signals from the hand were sufficient to differentiate between foot strike patterns.

## 3. Proposed Approach

In this section, we describe a wearable system and machine learning pipelines for learning features extracted from the MTS signals captured while walking and running with a set of machine learning algorithms.

### 3.1. Activity Definition

**Figure 1:**
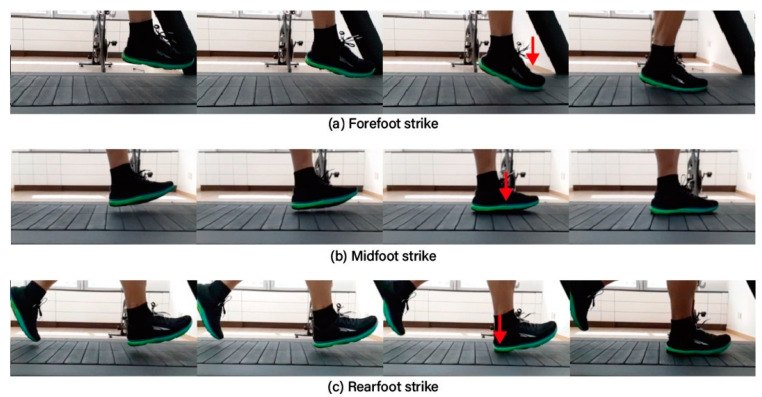
Three different striking patterns; (**a**) forefoot (FF) strike, (**b**) midfoot (MF) strike, (**c**) rearfoot (RF) strike. Red arrow marks the moments in which first contact is made on the ground.

As discussed in the previous section, we focused on three different foot striking strategies while running and walking, as shown in the figure below. FF strike or toe walking refers to the metatarsal heads of the foot touching the ground. In the case of an MF strike, the foot contacts the ground across the metatarsal head and subsequently contacts the ground. Although arguable, the MF strike pattern is considered to be more efficient and safer than RF because physical impacts are mitigated during the landing phase [47,48]. This type of striking strategy is often adopted by professional mid- and long-distance runners [48]. In the case of the RF strike, the so-called heel strike or heel-to-toe walking, the foot contacts the ground with the heel first. Figure 1 illustrates three types of striking strategies: FF, MF, and RF.

**Figure 2:**
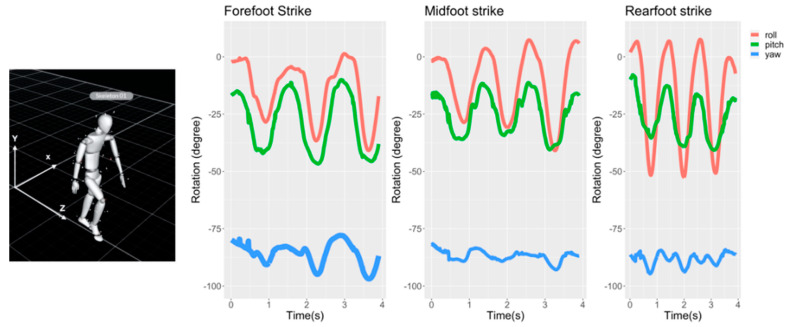
Motion tracking examples while walking with different FS types (**right**). Angular motions (roll, pitch, and yaw) of the (**left**) forearm are displayed.

As a preliminary study, we investigated whether the motion sequences of other body parts (e.g., hand) are affected by the FS patterns using a motion capture system. Using a smartwatch worn on the dorsal part of the left wrist, we visualized the angular movements (i.e., roll, pitch, and yaw) of the left forearm. Figure 2 shows the three-dimensional motions of 37 reflective markers tracked at a sampling rate of 240 Hz with a multi-camera motion capture system (NaturalPoint Inc., Corvallis, OR, USA) and examples of calculated angular motions (i.e., roll, pitch, and yaw) of the left hand.

As shown in Figure 2, different hand motions (e.g., arm swing patterns) were systematically observed with respect to the striking strategy. Based on this observation, we hypothesized that FS patterns could be differentiated using inertial measurements from the hand.

### 3.2. System for Data Collection

**Figure 3:**
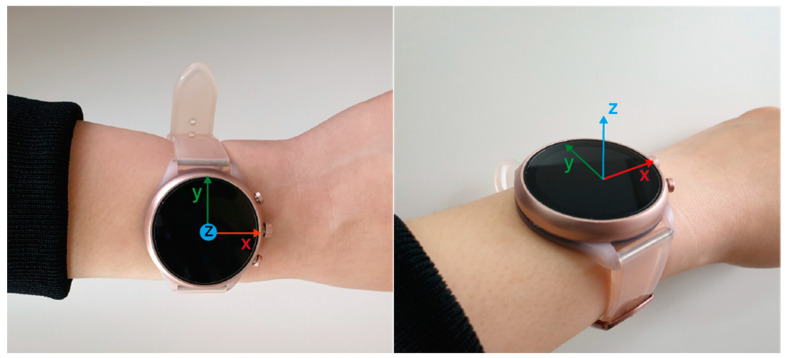
The smartwatch with its axis displayed adopted for the experiment.

We implemented a custom software for capturing inertial motions in the form of MTS signals (e.g., accelerations and angular velocities) for a commercial smartwatch (Sport Smartwatch FTW6024 by Fossil), which runs on Wear OS by Google. The sampling rate was set to 50 Hz, which was the fastest setting for the device. Figure 3 shows the smartwatch employed in this study, with its axis displayed.

### 3.3. Models

We adopted both feature-based machine learning and recent deep learning-based algorithms to learn the captured MTS data in a supervised setting.

#### 3.3.1. Baseline: Feature-Based Machine Learning

As the baseline, we employed a range of feature-based machine learning algorithms, such as naïve Bayes [49], random forest [50], and support vector machines [51].

The naïve Bayes classifier is a probabilistic machine learning model that uses the Bayes theorem [52]. This approach is simple yet well suited for many practical classification problems. However, its performance is often hindered by the naïve assumption that features are conditionally independent and contribute equally to the output. A random forest classifier utilizes a large number of decision trees in an ensemble on various subsamples of the dataset [53]. In particular, random forests demonstrate robust and reliable performance across many application fields. In addition, we utilized SVM [51], which is effective in classification problems in high-dimensional spaces, as a classifier. A radial basis function (RBF) kernel was used for SVM.

For feature-based approaches, we conducted extensive feature engineering work beforehand according to a recent work [54], which identifies the significant time-series characteristics statistically [55].

#### 3.3.2. End-to-End Machine Learning

To learn complex and hierarchical features from raw sensor signals in an end-to-end learning fashion, we adopted recent deep learning algorithms, such as Conv1D [29,30], gated RNNs, such as LSTM [56] and GRUs [28]. In particular, both LSTM and GRU typically outperform vanilla RNN (i.e., RNN with a traditional tanh unit) in many sensor-based applications [3,4,31,42,57,58,59,60] because these models can capture long-term dependencies efficiently by mitigating the vanishing gradient problem utilizing memory cells and/or gating units. Refer to [28] for further details.

## 4. Experiment

This section describes experiment procedures designed for the proposed sequence classification tasks.

### 4.1. Data Acquisition

This study was approved by the Institutional Review Board of the Hallym University (HIRB-2021-058). Written informed consent was obtained from all participants. Data were collected under three types of running and walking conditions. The experimenter provided instructions on which part of the foot hit the ground first to participants who walked or ran with different strike patterns. The participants had sufficient time before data acquisition to practice the desired for strike types. Based on visual inspection, the researcher provided feedback to adjust the target pattern for each subject. The participants were recruited from local universities. Most of the participants were recreational and elite athletes. In total, 1,279,500 data points (approximately 7.10 h) of walking data and 2,891,000 data points (approximately 16.06 h) of running data were collected. We screened and categorized the recorded data into three types of FS patterns by visual inspection.

#### 4.1.1. Walking

Sixteen participants (11 females) with a mean age of 31.06 ± 10.24 years and an average body mass index (BMI) of 21.89 ± 4.15 kg/m^2^ were asked to walk in a set of flat grounds including outdoor flat ground, an indoor corridor, and a treadmill at self-paced speeds with different walking strategies. As a reference, we also collected data from standstill postures, such as standing and sitting (e.g., doing something while sitting and standing) from four different participants. Table 1 shows the acquired results for foot strike patterns. The whole dataset was split into 70% and 30% for training and testing processes, respectively.

#### 4.1.2. Running

Seventeen participants (8 females) with a mean age of 28.18 ± 12.12 years and an average BMI of 20.49 ± 3.69 kg/m^2^ were asked to run tracks in sports facilities on a university campus, park, or treadmill at self-paced speeds with different striking strategies, similar to the walking condition. Table 2 shows the acquired results for foot strike patterns.

### 4.2. Classification

**Figure 4:**
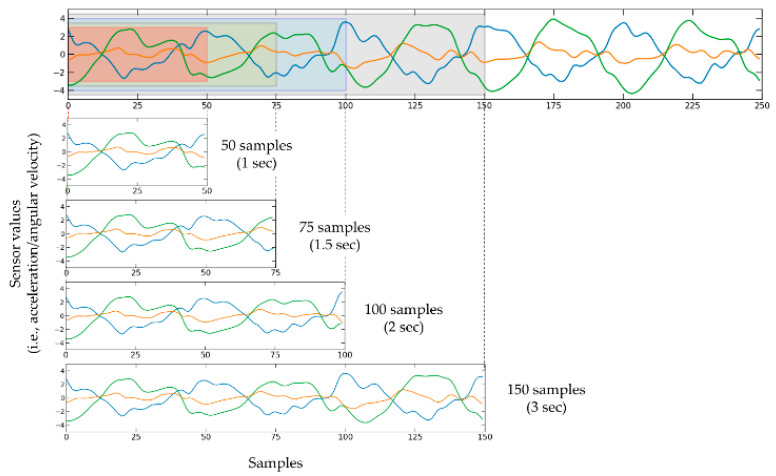
Example process of segmenting the raw measurements with different segmenting windows, highlighted for visualization purposes. Noting that inertial signals have periodic characteristics, the input 6-axis raw signals were partitioned into data segments (*L* × 6) with four different fixed-lengths of signals (*L* = 50, 75, 100, and 150 samples, each of which corresponds to approximately 1.0, 1.5, 2.0, and 3.0 s) for investigating the effective signal length of running and walking activities. Note that the beginning and end of a cycle of repetitive motion are not aligned with a segmenting window so that an arbitrary part of raw measurements can be segmented.

In this section, we evaluated whether the captured motion signals reveal sufficient information to differentiate the FS patterns. Raw sensor measurements were normalized by removing the mean and then scaling to unit variance on each axis for further feature engineering processes. Because walking and running, characterized by repeated movements, have different step cycles between activities and individuals, raw signals were partitioned into four different signal lengths (L = 50, 75, 100, and 150 samples, which correspond to approximately 1.0, 1.5, 2.0, and 3.0 s, respectively) with no overlaps between partitions. Therefore, the input to the system is a six-dimensional (6D) tensor, which represents the waveform data of four different fixed-length windows. Figure 4 illustrates an example signal with segmentation windows of different lengths, highlighted for visualization purposes.

#### 4.2.1. Feature-Based Classification

Once segmented signals were normalized, target features were extracted with the tsfresh package [54] with a predefined setting, which was set to calculate 781 predefined features from a given signal, resulting in 4686 (= 781 × 6 axis) aggregated features for each segmented input data. The extracted features were then filtered into a subset of significant features based on the feature selection algorithm [54], which evaluates the statistical significance of each time-series feature based on the Benjamini–Yekutieli procedure [55]. Statistical analysis of our dataset showed 2333 features out of 4686 to be significant, and we selected the 180 most significant features for each segmented input dataset.

**Figure 5:**
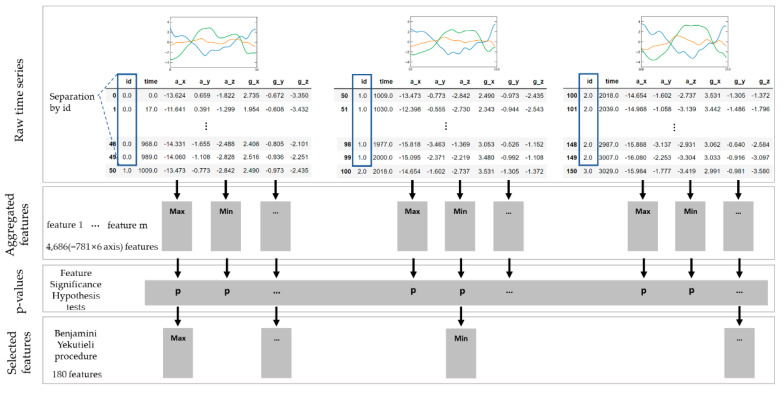
Workflow of feature filtering process. A set of features was extracted from the partitioned MTS data and then selected according to a statistical significant test [54].

Figure 5 illustrates the process of feature extraction, aggregation, and selection process. As classifiers for the feature-based approach, we utilized naïve Bayes (NB), random forest (RF), and support vector machine (SVM) classifiers.

Table 3 shows examples of selected significant features from the dataset.

#### 4.2.2. End-to-End Machine Learning

As described in the previous section, we employed a set of recent deep learning architectures for training the acquired MTS data in an end-to-end fashion: Conv1D [29,30], gated RNNs, such as LSTM [56] and GRUs [28].

**Figure 6:**
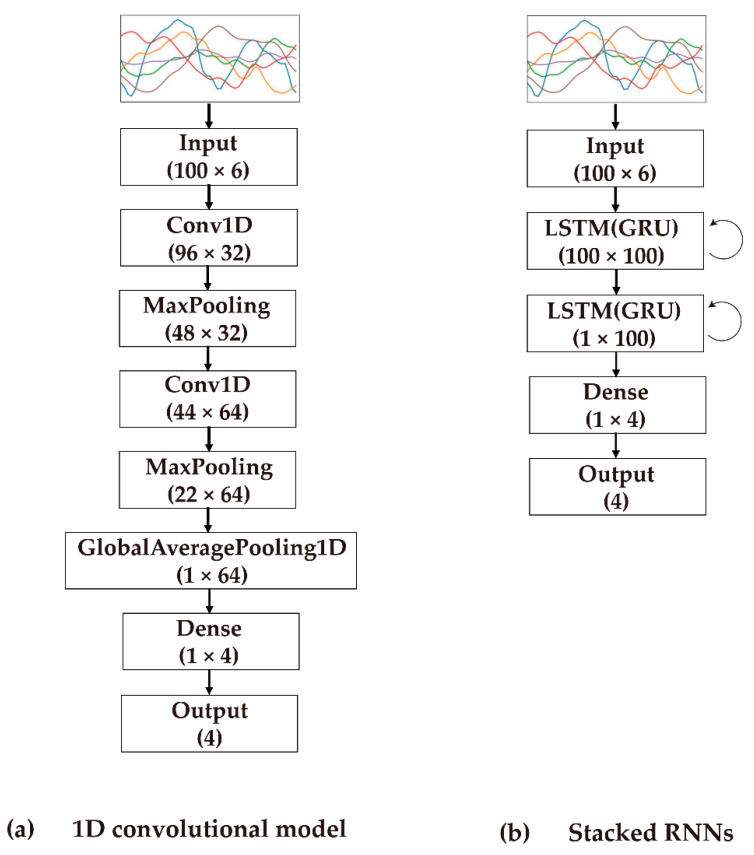
Network architecture used in this study. Here, the diagram above is based on input signal length of *L* = 100. In case of gated RNNs, LSTM/GRU cells are stacked to learn more complex representations.

For the Conv1D model shown in Figure 6a, we first obtained convolutional features by applying 1D convolution operations over one-dimensional input signals and aggregated the features from the previous layers by applying a max-pooling operation. These operations were repeated twice to extract and abstract the temporal features. The convolution operation was performed with a kernel size of 5, no padding, and a stride of 1. Finally, we utilized the global average pooling layer [61] to calculate average of each feature map instead of adding a fully connected layer on top of the flattened feature maps.

For the GRU- and LSTM-based models shown in Figure 6b, we stacked recurrent cells twice (i.e., stacked two-layer GRU/LSTM) to increase the depth of the network. The dimension of the recurrent hidden states was set to 100, which was identical to the length of the input signal, *L*.

For all neural-network-based models used in this study, dropout layers were applied to applied to the network to prevent the networks from overfitting. The last dense layer in the deep neural network used for the classification task has four neurons with a softmax activation function corresponding to the four different target motions: three types of foot strike patterns and one standby action. For the optimization process, we employed an ADAM optimizer [62].

Feature-based approaches, such as naïve Bayes, random forest, and SVM, were implemented with Scikit-Learn and end-to-end learning approaches were implemented with Keras using Tensorflow as the backend.

### 4.3. Results

We employed $F_{m}$, the weighted average of the F1-scores, as a performance metric as shown in Equation (1).

(1) $$F_{m}=2\overset{C}{\underset{c=1}{\sum }}\frac{N_{c}}{N_{tot}}\;\frac{Precision_{c}\times Recall_{c}}{Precision_{c}\times Recall_{c}}$$

Here, $N_{c}$ is the number of samples belonging to class C, and $N_{tot}$ is the total number of samples. Table 4 shows the experimental results of the proposed approach in terms of $F_{m}$.

The average elapsed times for inferencing 100 samples (= 100 × *L* × 6) using pretrained NB, RF, SVM, LSTM, GRU, and Conv1D classifiers are displayed according to the data types in Table 5. For feature-based learning, we extracted the 180 preselected features from each segment of the data (*L* × 6), determined during the training phase (see Table 3).

The normalized confusion matrix for classification results and two-dimensional embeddings of high-dimensional features using the t-distributed stochastic neighbor embedding (t-SNE) algorithm [63] are described in the Appendix A.

## 5. Discussion and Limitations

### 5.1. Classification Performance

Experimental results from feature-based approaches indicate that SVM achieved the best performance.

The $F_{m}$ of SVM was 93.927 for walking and 95.303 for running conditions. The $F_{m}$ of RF and NB were 83.899 (83.816) and 73.348 (72.277) for walking and 81.242 (78.082) and 50.250 (56.129) for the running condition, respectively. Note that RF and NB have difficulties not only in learning the training dataset but also in generalizing to the test dataset.

In the end-to-end deep learning approach, Conv1D achieved the best classification performance; the test $F_{m}$ was 96.379 for walking and 98.056 for running conditions. Regarding the convolutional operation, we experimented with kernel sizes of 3 and 5; however, we obtained similar accuracies: 95.898 for walking and 97.800 for running conditions when a convolution kernel size of 3 was used.

The test $F_{m}$ of GRU and LSTM were 94.409 and 94.064 for walking and 97.426 and 96.881 for the running condition, respectively. In summary, deep learning-based approaches generally demonstrated higher $F_{m}$ because of their capability to learn a hierarchical feature representation from raw MTS signals [64].

**Figure A1:**
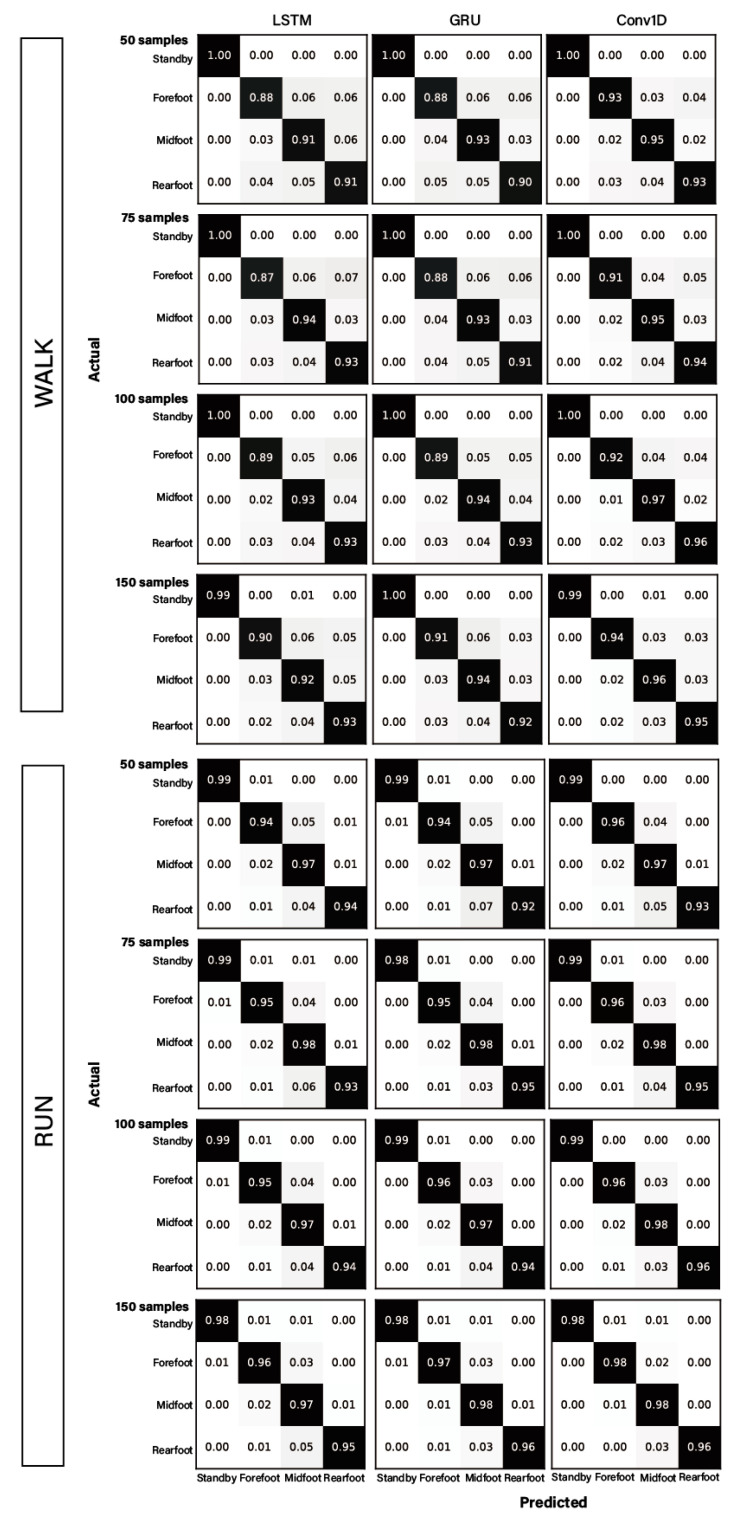
Normalized confusion matrices when deep neural network-based classifiers were employed for classifying the walking data and running data.

**Figure A2:**
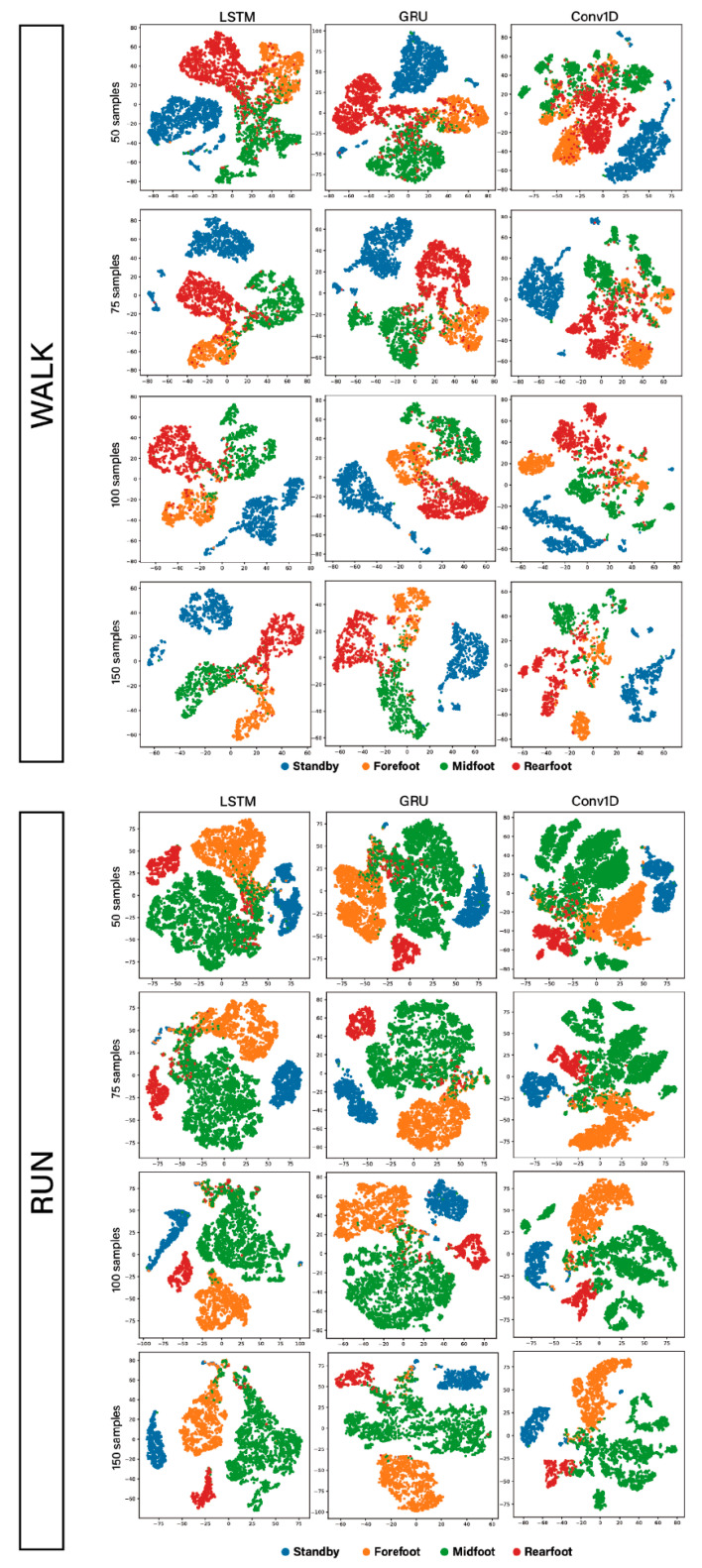
Visualization of internal features using t-SNE (employing all the test data from the walking and running condition). As a nonlinear dimensionality reduction technique, t-SNE was applied in Figure A2. Each 2D point represents the projected feature embedding of a data segment of approximately 1 s.

Figure A2 shows the two-dimensional embeddings of high-dimensional feature space (64 for Conv1D and 100 for LSTM and GRU in our case) for the walking and running conditions, respectively, using the t-SNE algorithm, which projects points in a high-dimensional space into a 2D space in such a way that similar points cluster together [63]. Here, each 2D point represents the projected feature embedding of a data segment containing 1 to 3 s. As shown in the figures, feature embeddings are well clustered in separate regions, meaning that fine-grained motion features from the different FS conditions were successfully learned in an end-to-end manner. In particular, for standby motion, the t-SNE plots showed a more clustered distribution. Consequently, standby motion represents a more accurate classification of the results, as shown in Figure A1.

### 5.2. Data Imbalance

Furthermore, although the dataset from the running condition is imbalanced as RF cases have fewer observations than the other cases (i.e., 12.42% compared to the dataset from the MF strike condition), the experimental results showed that deep learning-based approaches exhibited high $F_{m}$ of 98.056 for Conv1D, 97.426 for GRU, 96.881 for LSTM. In addition, as Figure A2 shows, two-dimensional feature embedding of the test sets of the RF striking condition (shown in red) are well separated from other classes, indicating that our system extracted and learned features from the imbalanced MTS data successfully.

### 5.3. Effect of Waveform Length

As shown in Table 4 and Figure A1, the performance of the machine learning models was influenced by the length of the input waveform. In our results, the performance was generally enhanced as the sample length increased. This is generally consistent with the results of previous studies that examined the effects of the sample length on the performance of the suggested systems [65,66]. Interestingly, sample lengths with the highest performance in walking and running were shown to be slightly different; signal lengths of 100 samples (approximately 2 s) and 150 samples (approximately 3 s) demonstrated the best $F_{m}$ for most classifiers in both walking and running conditions. Considering that the time required for RNN-based approaches during the inference phase increases as the input length increases owing to their recurrent nature, as shown in Table 5, the Conv1D-based approach is advantageous in terms of securing performance and reducing computational time, especially when longer parts of MTS signals are considered. Based on the experimental results obtained, we can conclude that foot-striking patterns can be estimated from the smartwatch, as we hypothesized, although performance varies depending on data segmentation methods. We believe that the proposed smartwatch-based recognition would help users to exercise unintrusively and effectively, although a similar purpose can be achieved through direct measurements from the foot, as in [36,37,46]. In a similar context, we plan to extend the proposed approach using other types of wearable devices (e.g., mobile phones in the pocket and necklace-shaped devices).

### 5.4. Applications & Explorations

An intelligent system that detects FS patterns in real time is beneficial for many sports training applications. For example, a wearable smartwatch device may inform runners when they are running with repetitive RF striking patterns, preventing continuous exposure to risk factors for musculoskeletal injuries.

In another setting, our system can be used as a new health-monitoring feature. For example, once unusual striking patterns (e.g., walking waddlingly or walking with a limp) are detected while walking, the system can provide users or their family members with informed alarms since individual variability of walking patterns can be a warning sign of health problems.

### 5.5. Limitations and Future Work

#### 5.5.1. Inter-User Variability

**Figure A3:**
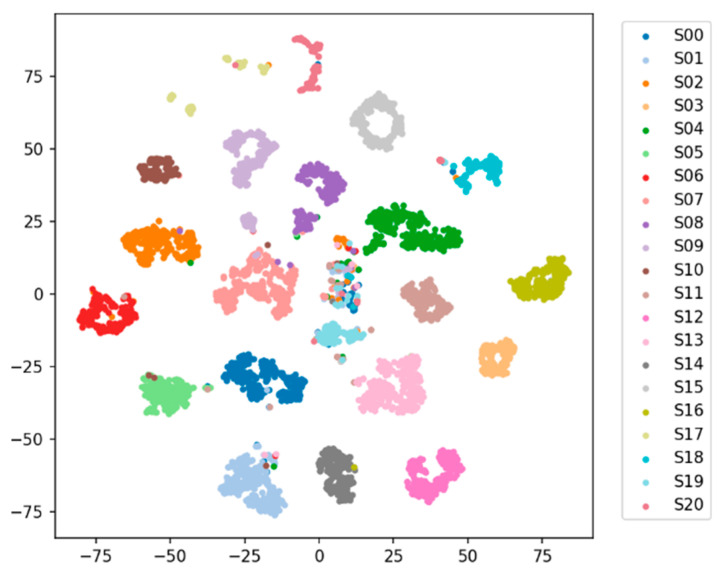
Visualization of internal features for recognizing the users when a GRU-based classifier is applied using t-SNE. Each color represents a different participant.

Throughout this study, we focused on extracting and learning user-independent features from the MTS motion signals for the proposed classification task. After conducting a further classification test, which evaluates whether the captured MTS signals contain sufficient information to differentiate between participants. As shown in Figure A3, deep neural networks (i.e., GRU) can successfully differentiate a specific user from others. Interestingly, the experimental results demonstrated that deep learning-based approaches exhibited high test accuracy ($F_{m}$ score) of 97.871 (97.874) for Conv1D, 97.075 for GRU, and 97.159 for LSTM for the running dataset. We expect that inter-user variability can be utilized for opening a new venue for promising future work: recognition of athletes and their style changes over time.

#### 5.5.2. Scalability to a Large Dataset

Because we collected data from a limited number of participants (16 for walking; 17 for running except the standby posture), we acknowledge that models trained with the current dataset cannot be readily applicable to classify the data from arbitrary participants. For the model to be more generalizable, we plan to collect datasets from diverse participants.

## 6. Conclusions

In this paper, we have presented a novel wearable system that can estimate FS patterns using inertial measurements from a smartwatch, hypothesizing that walking and running styles affect not only the pressure distribution on the foot but also whole-body movements. To evaluate this hypothesis, we first collected MTS signals using a commercial smartwatch with custom software and then trained the dataset with a set of supervised learning models, which included feature-based and recent deep learning-based architectures. The experimental results validated the feasibility of the proposed approach. The deep learning-based approach exhibited high performance; The $F_{m}$ of the Conv1D-based model was 96.379 for classifying the walking data, and 98.056 for the running data, while feature-based approaches demonstrated lower classification performance despite the extensive feature engineering process.

Because our experimental results demonstrate that motion signals captured from the hand reveal enough information to differentiate subtle walking and running styles, we believe that the proposed intelligent system can be utilized for monitoring the quality of motion in an unintrusive and natural manner.

In summary, we expect that the proposed approach would inspire a variety of intriguing HAR applications in which only indirect motion consequences are measurable.

## Figures and Tables

**Table 1:** Data acquired while walking with different strategies.

	Total Duration (min)	Time per Subject (Mean ± SD)
Standby	144.45	35.83 ± 7.67
Forefoot strike	80.16	7.13 ± 12.21
Midfoot strike	116.57	9.38 ± 16.18
Rearfoot strike	107.95	9.39 ± 16.07

**Table 2:** Data acquired while running with different strategies.

	Total Duration (min)	Time per Subject (Mean ± SD)
Standby	319.3	79.38 ± 59.84
Forefoot strike	270.21	29.36 ± 46.25
Midfoot strike	529.96	38.86 ± 47.78
Rearfoot strike	65.82	10.83 ± 5.67

**Table 3:** Examples of selected common features based on the feature significance test. Feature names were adopted from [54].

Feature Name	Descriptions
change_quantiles	the average, absolute value of consecutive changes of the time series inside the corridor
cwt_coefficients	a continuous wavelet transform for the Ricker wavelet, also known as the “Mexican hat wavelet”
fft_coefficient	the Fourier coefficients of the one-dimensional discrete Fourier Transform for real input by fast Fourier transformation algorithm
agg_linear_trend	a linear least-squares regression for values of the time series
quantile	the q quantile of time series
permutation_entropy	the permutation entropy
autocorrelation	the autocorrelation of the specified lag
ar_coefficient	the unconditional maximum likelihood of an autoregressive process
fourier_entropy	the binned entropy of the power spectral density of the time series
number_peaks	the number of peaks of the time series
fft_aggregated	the spectral centroid (mean), variance, skew, and kurtosis of the absolute Fourier transform spectrum
ratio_beyond_r_sigma	ratio of values that are more than r ∗ std (time series) away from the mean of time series
agg_autocorrelation	the autocorrelation of the time series
partial_autocorrelation	the value of the partial autocorrelation function at the given lag
spkt_welch_density	the cross power spectral density of the time series at different frequencies

**Table 4:** Experimental results of test *F_m_*.

Type	Signal Length	Feature-Based Learning			Deep Neural Network		
		NB	RF	SVM	LSTM	GRU	Conv1D
walk	50	68.220	78.042	90.103	92.880	93.189	95.536
	75	68.929	80.282	92.305	93.847	93.378	95.436
	100	71.197	80.964	93.469	94.306	94.280	96.379
	150	72.277	83.816	93.927	94.064	94.409	96.313
run	50	47.014	74.896	93.578	96.270	96.050	96.772
	75	51.495	77.820	94.493	96.704	96.715	97.150
	100	57.143	78.465	94.632	96.720	97.013	97.596
	150	56.129	78.082	95.303	96.881	97.426	98.056

**Table 5:** Average elapsed times in milliseconds for inferencing test data with a batch size of 100. For the feature-based learning, elapsed times for feature extraction are included. Average inference time is displayed in parentheses after individual numbers.

Type	Signal Length	Feature-Based Learning			Deep Neural Network		
		NB	RF	SVM	LSTM	GRU	Conv1D
walk	50	145.54(3.02)	153.22(9.23)	149.56(4.42)	35.34	36.39	27.70
	75	139.91(3.13)	141.74(9.47)	135.13(3.83)	38.51	38.41	27.58
	100	131.48(3.13)	138.24(9.43)	131.86(3.37)	42.14	41.49	28.10
	150	127.52(3.09)	132.93(9.52)	129.17(3.39)	46.53	46.00	28.95
run	50	143.64(3.03)	150.45(9.39)	145.59(5.04)	35.89	36.27	28.39
	75	147.81(3.09)	154.36(9.38)	147.26(4.26)	38.42	38.78	28.18
	100	141.39(2.98)	151.23(9.50)	145.17(4.06)	41.58	41.56	27.82
	150	130.96(3.11)	139.13(9.42)	134.80(3.70)	46.94	46.31	28.10
